# Global and regional source attribution of Shiga toxin-producing *Escherichia coli* infections using analysis of outbreak surveillance data

**DOI:** 10.1017/S095026881900116X

**Published:** 2019-07-08

**Authors:** Sara M. Pires, Shannon Majowicz, Alexander Gill, Brecht Devleesschauwer

**Affiliations:** 1 National Food Institute, Technical University of Denmark, Lyngby, Denmark; 2 University of Waterloo, Waterloo, Ontario, Canada; 3 Bureau of Microbial Hazards, Health Canada, Ottawa, Ontario, Canada; 4 Department of Epidemiology and Public Health, Sciensano, Brussels, Belgium; 5 Department of Veterinary Public Health and Food Safety, Faculty of Veterinary Medicine, Ghent University, Brussels, Belgium

**Keywords:** Food safety, outbreaks, Shiga toxin-producing *Escherichia coli* (STEC), source attribution

## Abstract

Shiga toxin-producing *Escherichia coli* (STEC) infections pose a substantial health and economic burden worldwide. To target interventions to prevent foodborne infections, it is important to determine the types of foods leading to illness. Our objective was to determine the food sources of STEC globally and for the six World Health Organization regions. We used data from STEC outbreaks that have occurred globally to estimate source attribution fractions. We categorised foods according to their ingredients and applied a probabilistic model that used information on implicated foods for source attribution. Data were received from 27 countries covering the period between 1998 and 2017 and three regions: the Americas (AMR), Europe (EUR) and Western-Pacific (WPR). Results showed that the top foods varied across regions. The most important sources in AMR were beef (40%; 95% Uncertainty Interval 39–41%) and produce (35%; 95% UI 34–36%). In EUR, the ranking was similar though with less marked differences between sources (beef 31%; 95% UI 28–34% and produce 30%; 95% UI 27–33%). In contrast, the most common source of STEC in WPR was produce (43%; 95% UI 36–46%), followed by dairy (27%; 95% UI 27–27%). Possible explanations for regional variability include differences in food consumption and preparation, frequency of STEC contamination, the potential of regionally predominant STEC strains to cause severe illness and differences in outbreak investigation and reporting. Despite data gaps, these results provide important information to inform the development of strategies for lowering the global burden of STEC infections.

## Introduction

Strains of *Escherichia coli* that produce Shiga toxins (Shiga toxin-producing *E. coli*, STEC) are an important cause of foodborne disease worldwide. The World Health Organization (WHO) recently estimated that foodborne STEC caused more than 1 million illnesses, resulting in more than 100 deaths and nearly 13 000 disability-adjusted life years (DALYs) in 2010 [1]. STEC infections have been associated with a wide range of illnesses, from mild intestinal discomfort to haemolytic uremic syndrome (HUS) or end-stage renal disease (ESRD) and death [2, 3]. Ruminants, including cattle, sheep, goats and deer, have been identified as the most important sources of this pathogen [4, 5]. STEC have also been isolated from a wide range of domestic and wild mammals, birds and insects; these animals may be less significant sources of STEC but play an important role in their distribution [6]. STEC infection is generally not associated with illness in animal hosts and thus STEC can be viewed as part of the natural microbiota of many animals, including ruminants.

Human STEC infection requires ingestion and transmission can occur through food, water, direct contact with animals, person to person contact and through fomites [7–9]. Knowledge of the contribution of these different sources and transmission routes for disease is essential to prioritise food safety interventions and implement appropriate control measures to reduce the burden of diseases in a population. However, information on the relative importance of different sources for STEC infections at regional or global levels is currently only available from expert elicitation studies [10–13].

A variety of methods to attribute human foodborne illnesses to the responsible sources have been developed during recent years [14] and several have been applied to investigate foodborne hazards in a wide range of populations. While source attribution of very common foodborne pathogens such as *Salmonella* and *Campylobacter* has been possible in several national and international studies [15–24], estimating the relative contribution of sources for other diseases has been difficult due to substantial data gaps. Among these is STEC, for which a very limited number of national studies have been conducted [5, 25]. These data limitations are particularly felt in terms of animal and food monitoring, whereas human disease surveillance data are available from multiple countries worldwide. Epidemiological approaches for source attribution use public health surveillance data to estimate the relative contribution of different sources, routes of exposure or risk factors for disease [14]. These include analysis of data from outbreak investigations, which have been used for source attribution of several pathogens [16, 21, 26, 27]. A simple summary of outbreak investigation results can be useful for identifying the most common foods causing human illness by a pathogen. However, often the implicated food is a ‘complex’ food, i.e. containing several food items and ingredients, where in principle any of them could be the specific source of the outbreak [26]. The objective of this study was to estimate the relative contribution of different foods for STEC infections globally and at the regional level. We applied a method based on outbreak data that is able to consider complex foods to attribute human STEC infections to specific sources in WHO regions and globally. This study was conducted under the umbrella of the Joint Food and Agriculture Organization (FAO)/World Health Organization (WHO) Core Expert Group on STEC/VTEC, which applied different approaches for source attribution of STEC illness [28].

## Methods

### Data

A call for STEC outbreak surveillance data was sent by WHO to national Codex contact points and via other relevant channels to Member Countries in April 2016 (http://www.who.int). The text of the call for data and a list of recipients are included in Supplementary Material 1. The request aimed at collecting data on all STEC outbreaks reported globally and had no time-period restriction. A reminder was sent in July 2017 and that the last data used were received in November 2017. Data received included both publicly available reports and datasets and grey literature reports. All data providers gave permission to utilise the data and publish results. Collected data were organised so that each reported outbreak corresponded to one observation in the final dataset. Each observation contained information on the year of occurrence, country, aetiology, number of ill people, number of HUS cases and fatalities associated with the outbreak, location of the outbreak and implicated source. All data reported as non-foodborne (e.g. waterborne outbreaks, person-to-person) were excluded from the dataset.

### Food categorization

To categorise foods, we applied the scheme produced by the US' Interagency Food Safety Analytics Collaboration [29], allowing for potential adaptations to accommodate sub-categories that are common in different countries or regions. The level of sub-categorisation within each main food category varied for different fields. As an example, while for under ‘land animals’, the lowest level of sub-categorisation was used, all fruits and all vegetables were grouped in the higher level category ‘produce’, respectively. Type of processing or degree of cooking (i.e. raw, undercooked, well-done) were not included in the categorisation scheme.

### Model overview

The method used in this study was based on a previously published method [27], modified and applied to the STEC dataset. The principle is to attribute human illnesses to food sources on the basis of the number of outbreaks that were caused by each of these foods. For this purpose, implicated foods are classified by their ingredients as simple (i.e. belonging to one single food category), or complex (i.e. belonging to multiple food categories). The ingredients that constitute the complex foods are designated through defined criteria [30]. The proportion of disease that can be attributed to each food source was estimated in a two-step process based on (a) the number of simple-food outbreaks caused by that source and (b) the number of complex-food outbreaks, the ingredients (food categories) composing complex-foods and the probability that each of these categories was the cause of the complex-foods outbreaks. The attributable proportions were calculated by WHO region (AFR: African region; AMR: Region of the Americas; EUR: European region; EMR: Eastern Mediterranean region; SEAR: South-East Asian region; WPR: Western Pacific Region).

In the first step, the number of simple-food outbreaks attributed to each single food category was calculated by WHO region. In the second step, we first calculated the probability *P*_*j*_ that an outbreak was caused by source *j*, by summarising the data from simple-food outbreaks per source across all countries and the whole study period. Specifically, *P*_*j*_ was defined as the proportion of single-food outbreaks caused by source *j*. The uncertainty in the probability vector ***P*** was quantified using a Dirichlet(***S***) distribution, with ***S*** the vector of components *S*_*j*_ corresponding to the number of single-food outbreaks caused by source *j*. Next, complex-food outbreaks were partitioned to each of the food categories in the implicated food proportionally to the probability *P*_*j*_ of causing a simple-food outbreak. We used a Monte Carlo simulation approach to propagate the uncertainty in *P*_*j*_ and to model the uncertain allocation of a complex-food outbreak to a specific food category. First, we simulated 10 000 values of *P*_*j*_ for each source *j*. Then, we multiplied *P*_*j*_ with a dummy matrix *F*_*ij*_, representing the implicated food categories *j* in outbreak *i*. As an example, outbreaks caused by *chilli con carne* would be attributed to the categories ‘beef’, ‘vegetables’, ‘grains and beans’ and ‘oils and sugar’; *F*_*ij*_ would thus contain the value 1 for sources ‘beef’, ‘vegetables’, ‘grains and beans’, and ‘oils and sugar’ and value 0 for all other sources. By multiplying with *P*_*j*_, outbreaks due to a complex food were only attributed to categories that had been implicated in at least one simple-food outbreak. In our example above, if ‘grains and beans’ and ‘oils and sugars’ were not implicated in any pathogen-specific outbreak caused by simple foods, these two categories would be excluded for the attribution of the *chilli con carne* outbreak. In the second step of the Monte Carlo process, we accounted for the uncertain allocation of complex-food outbreaks to specific food categories. For each complex-food outbreak *i* and per iteration of *P*_*j*_, we simulated 20 realizations of a multinomial distribution with size 1 and probability vector ***PF***_*i*_. For each complex-food outbreak *i*, this then resulted in 200 000 random attributions to a single source *j*. Finally, the results of the simple-food outbreaks were summed with the probabilistic attributions of the complex-food outbreaks, to obtain the total number of outbreaks, by region, attributed to each source:

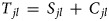

where *T*_*jl*_ is the total number of outbreaks attributed to source *j* in region *l*, *S*_*jl*_ the number of simple-food outbreaks caused by source *j* in region *l* and *C*_*jl*_ the number of complex-food outbreaks attributed to source *j* in region *l*. The proportion of disease attributed to each source *j*, again by region, was then obtained by dividing the total number of attributed outbreaks to the total number of reported outbreaks *T*_*l*_:

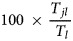

The resulting uncertainty distribution was summarised by its mean and a 95% uncertainty interval (UI) given by the distribution's 2.5th and 97.5th percentiles.

The number of ill people implicated in the outbreaks was not considered in the analysis to avoid potential overestimation of the importance of sources that caused large outbreaks. To estimate the relative importance of the food sources implicated in cases of HUS, we applied the same modelling approach to attribute the outbreaks that included HUS cases to food sources and the outbreaks that did not involve HUS cases (for comparison). In addition, to estimate relative importance of the food sources for severe cases of disease, we applied the same approach to outbreaks associated with fatalities. All models were implemented in R 3.5.1 [31].

## Results

### Data used in the model

STEC outbreak data were received from 27 countries covering the period between 1998 and 2017 and spanning three WHO geographic regions: AMR, EUR and WPR (Supplementary material 2). The oldest data reported were for outbreaks in the USA and covered the period from 1998 to 2015; the remaining countries reported data corresponding to outbreaks that occurred between 2010 and 2017.

In total, the dataset included 957 STEC outbreaks, the large majority (78%: 746/957) reported in the AMR. Of the 957 outbreaks, 361 (38%) were caused by a simple food, 57 (6%) by a complex food and 539 (56%) were caused by an unknown source (Table 1).

**Table 1.** Number and proportion of outbreaks caused by simple, complex or unknown foods in World Health Organization regions

	Simple		Complex		Unknown		
Region	Number	%	Number	%	Number	%	Total
AMR	283	38	61	8	402	54	746
EUR	55	31	14	8	107	61	176
WPR	7	20	4	11	24	69	35
Total	345	36	80	8	532	56	957
Outbreaks associated with HUS cases							
AMR	92	53	15	7	121	40	228
EUR	1	100	0	0	0	0	1
WPR	3	43	0	0	4	57	7
Total	96		15		125		236
Outbreaks associated with deaths							
AMR	21	50	1	2	20	48	42
EUR	2	100	0	0	0	0	2
WPR	1	100	0	0	0	0	1
Total	24		1		20		45

AMR, Region of the Americas; EUR, European Region; WPR, Western Pacific Region.

A total of 236 outbreaks that involved HUS cases were reported in the time whole period, nearly all (97%) in the AMR. Of these outbreaks reported in the AMR, 53% were caused by simple foods, 7% by complex foods and 40% by an unknown source (Table 1). Twenty-nine percent (276/957) of all reported outbreaks were associated with either HUS or deaths.

Most of the 45 outbreaks that involved fatalities were also reported in the AMR, the large majority of them being caused by simple foods (50%) or unknown source (48%) (Table 2).

**Table 2.** Proportion of STEC cases attributed to foods in World Health Organization Regions (%, mean and 95% uncertainty interval (UI)).

	AMRO			EURO			WPRO		
	Mean	95% UI		Mean	95% UI		Mean	95% UI	
Beef	18.3	17.8	18.6	11.8	10.8	13.1	2.7	0	2.9
Produce	16.1	15.5	16.5	11.4	10.2	12.5	13.6	11.4	14.3
Dairy	5.5	5.2	5.9	6.2	6.2	6.2	8.6	8.6	8.6
Grains and beans	1.4	1.1	1.7	1.2	1.1	1.7	0.4	0	2.9
Pork	1.2	1.1	1.5	1.7	1.7	1.7	1.6	0	5.7
Meat	1.1	1.1	1.3	2.3	1.7	2.8	1.7	0	5.7
Game	0.5	0.5	0.7	0.6	0.6	0.6	2.9	2.9	2.9
Lamb	0.4	0.4	0.5	0.6	0.6	1.1	0	0	0
Seafood	0.4	0.4	0.4	1.7	1.7	1.7	0	0	0
Nuts	0.4	0.4	0.4	0	0	0	0	0	0
Chicken	0.1	0.1	0.3	0	0	0.6	0	0	0
Eggs	0	0	0.1	0.6	0.6	0.6	0	0	0
Poultry	0	0	0	0	0	0	0	0	0
Ducks	0	0	0	0	0	0	0	0	0
Turkey	0	0	0	0	0	0	0	0	0
Mutton	0	0	0	0	0	0	0	0	0
Oils and sugar	0	0	0	0	0	0	0	0	0
Unknown	54.4	54.4	54.4	61.9	61.9	61.9	68.6	68.6	68.6

AMR, Region of the Americas; EUR, European Region; WPR, Western Pacific Region.

### Attribution to foods

**Fig. 1.**
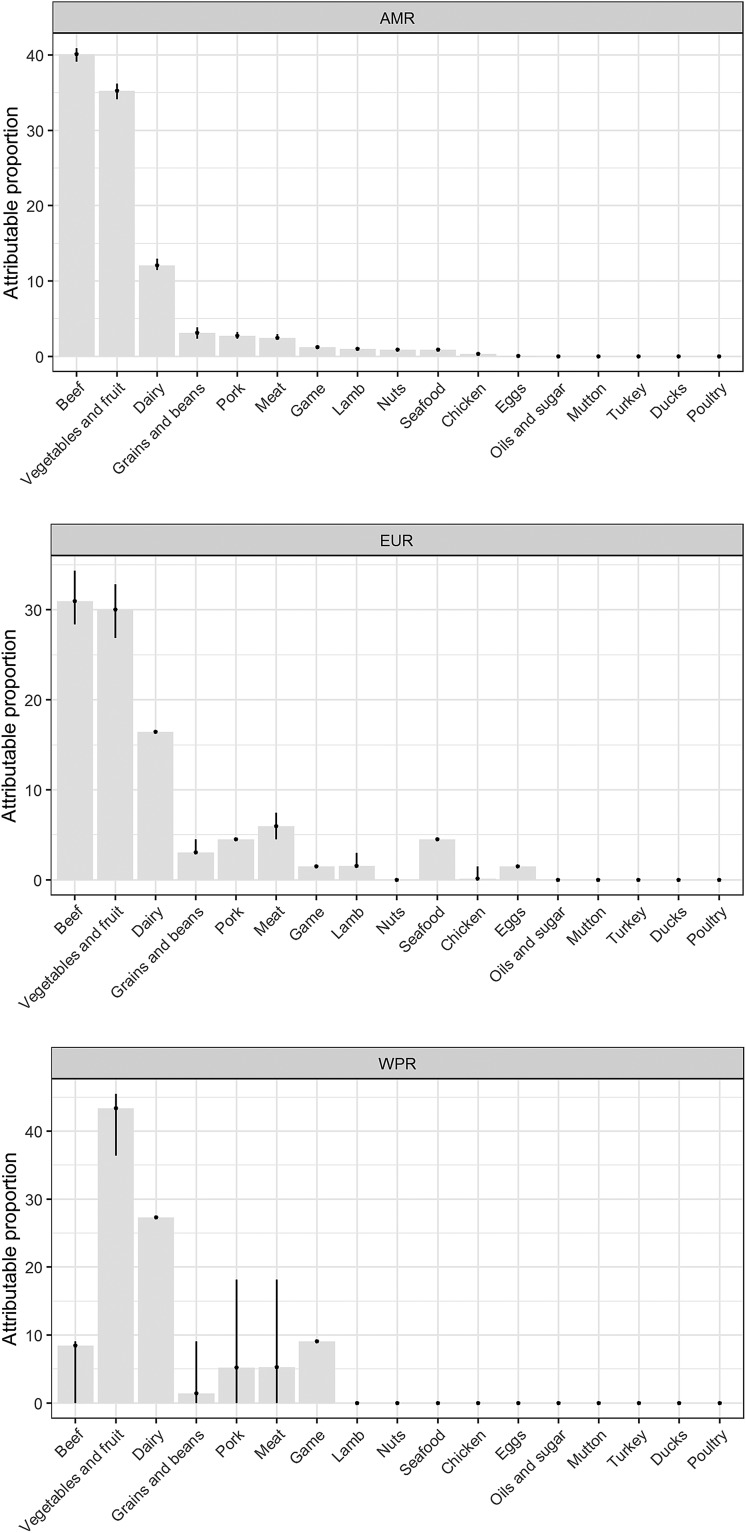
Relative contribution of foods categories to STEC cases in WHO regions (mean %). Estimates exclude proportion of unknown-source outbreaks. *AMR: Region of the Americas; EUR: European Region; WPR: Western Pacific Region.

**Fig. 2.**
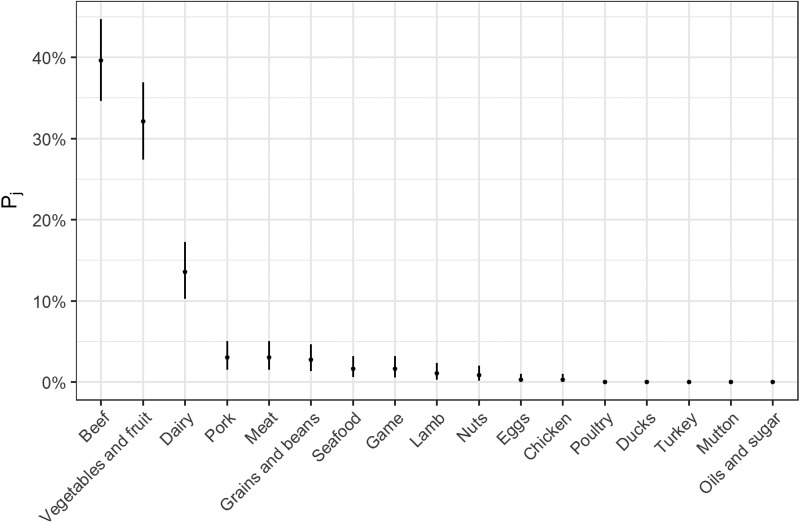
Estimates for probability that a complex-food outbreak was caused by source j (Pj) (median and 95% uncertainty interval).

Our results show that WHO regions differed in the relative contributions of different sources of STEC (Table 2, Fig. 1). When outbreaks attributed to *unknown source* were excluded (56% in the overall dataset), beef and produce were responsible for the highest proportion of cases in the AMR, 40% (95% UI 39.1–40.9%) and 35% (95% UI 34.1–36.2%), respectively (Fig. 2); all following estimates disregard outbreaks attributed to *unknown source*. Twelve percent (95% UI 11.5–12.9%) of STEC cases in AMR could be attributed to dairy products. In the EUR, the ranking of the sources of cases was similar though with less marked differences between each source, with an overall attribution proportion of 31% (95% UI 28.4–34.3%) for beef, 30% (95% UI 26.9–32.8%) for produce and 16% for dairy. In contrast, the most common source of STEC in WPR was produce (43%; 95% UI 36.4–45.5), followed by dairy (27%) and with game and beef third and fourth (9% and 8% (95% UI 0–9.1), respectively). It is important to note that in this region approximately 5% (95% UI 0–18.2%) of outbreaks with known source were attributed to another category ‘meat’, which cannot distinguish between the relative contributions of different animal sources. Among all other meat categories, pork played a minor role, with an attribution proportion between 3 and 5% across regions. The general term ‘poultry’, turkey, or ducks was never cited as a source of any outbreaks in any region; however, chicken was mentioned as a source in a very few outbreaks in the AMR (0.3%, 95% UI 0.3–0.6%) and the EUR (0.1%, 95% UI 0–1.5%). The proportion of STEC outbreaks attributed to an unknown source varied between 54% in AMR and 69% in WPR. Because data were only available from three out of six WHO regions, it was not possible to estimate global STEC source attribution proportions.

The estimates of the probability that a complex-food outbreak was caused by source *j* (*P*_*j*_) are plotted in Fig. 2. Results show that beef, produce and dairy were the sources with highest probability of being the cause of an STEC outbreak caused by a complex food. In other words, for example if a complex food containing beef, grains, dairy and eggs was implicated in an outbreak, the probability that it was caused by beef was 70% (95% UI 64–77%), by grains 5% (95% UI 2–8%), by eggs 0.05% (95% UI 0–0.02%) and by dairy 24% (95% UI 19–30%).

To estimate the relative contribution of different food sources for severe STEC cases, we restricted the analysis to data from AMR, where most of the outbreaks involving HUS cases or deaths were reported. We found no significant differences between attribution proportions for mild and severe disease (Supplementary material 3).

## Discussion

Our results show that the most important food types identified as sources of globally documented outbreaks caused by STEC were produce, beef and dairy products. The ranking of the top three food categories varied between regions. The proportion of STEC cases estimated to be attributable to beef and produce were highest in the AMR and EUR regions. In WPR, dairy appeared to play a more important role, followed by produce; beef ranked third. Possible explanations for regional variability include differences in the proportion of specific foods in the diet and how they are prepared for consumption, the frequency of STEC contamination of foods and differences in how outbreaks are investigated and reported. An additional potential source of variability between regions is differences in the prevalence of STEC strains with the potential to cause severe illness, such as bloody diarrhoea or HUS. Cases of severe illness are more likely to be reported and investigated and the potential to cause severe illness is variable between STEC strains [32, 33]. Both the predominant STEC serotypes and the genotypes within serotypes have been reported to vary between geographic regions [33–38]. More than half of the outbreaks documented globally could not be attributed to any source.

The data included in this analysis covered a broad time period (1998–2017) and we did not account for possible changes in pathogen incidence, outbreak surveillance, or illness attribution over time. As these factors and food preferences change over time, attribution estimates may change. The association of specific food categories with STEC illness reflects the historical practices of food production, distribution and consumption. Changes in production, distribution and consumption may result in changes in STEC exposure. Consequently, microbial risk management should be informed by an awareness of current local sources of STEC exposure.

To investigate the relative contribution of different sources for severe cases of disease, we restricted the analysis to outbreaks leading to cases of HUS or to deaths. Due to limited data availability, these analyses were restricted to the AMR. No substantial differences were identified in the attribution proportions for milder cases, HUS cases or deaths.

Our estimates show that a few food categories are responsible for a large proportion of STEC illnesses at the global level (produce, beef and dairy). We should note that the analysis grouped implicated food categories and that results do not suggest that all food items within these large categories are frequent sources of STEC. As an example, ‘produce’ includes a wide range of vegetable products and STEC outbreaks have been frequently linked to a few food items within this group (e.g. lettuce, spinach [39, 40]). Additionally, the frequency of a food as a source of STEC is not necessarily proportional to the frequency with which it is contaminated with STEC. For example, a 3-year study from the USA reported that the frequency of STEC contamination of romaine lettuce was 0.02% for STEC O157 and 0.09% for other STEC (200 g samples *n* = 5548) and that the frequency in spinach was similar (0.02% for STEC O157 and 0.09% for other STEC; 200 g samples *n* = 5325) [41]. For comparison, the frequency of six serogroups of non-O157 STEC in domestic raw ground beef components samples from USA federally inspected plants tested in 2012 was 2% (325–375 g samples *n* = 2119) [42]. A US National survey of bulk tanker milk reported an even higher frequency of STEC contamination, with 14% of samples (100 ml samples, *n* = 234) testing positive for Shiga toxin genes [43]. This discrepancy can be explained by the role of factors like the type of food processing and the degree of cooking in determining the likelihood of contamination of a food product at consumption. Still, the limited number of categories identified as important suggests that interventions for STEC focusing on these three food categories may be most effective in reducing illnesses [44]. These results can help identify the specific commodity producers and processors that regulators should engage and priorities for research on pre-harvest and post-harvest interventions to prevent or mitigate STEC contamination.

The data-driven source attribution estimates presented are based on data from outbreak surveillance. The overall assumption of this model is that the estimated attribution proportions based on outbreak data can be used to attribute the overall burden of STEC infections (i.e. the total incidence, including both outbreak-associated and sporadic cases) [16, 26]. However, there are a number of uncertainties linked to this assumption. Firstly, some foods are more likely to cause outbreaks than others and especially large outbreaks; thus, the relative importance of sources of outbreak-associated cases may not be representative of the overall contribution of sources for the total burden of disease [14]. The estimated relative contribution of each food type is dependent upon the probability that the food is involved in outbreaks that are identified and successfully investigated. For example, cases of severe illness or illness in children tend to be more frequently notified and cases of young adults less frequently [45]; this may also be true for outbreaks. Thus, certain risk groups within the larger population and smaller outbreaks may be underrepresented in the available data and more data are required to improve these estimates. Overall, estimates inevitably depend on the likelihood of a food being investigated in an outbreak, as well as the reporting capacity of each country. To avoid potential overestimation of the importance of sources that have caused a small number of large outbreaks the number of ill people implicated in the outbreaks was not considered in the analysis. Also, foods identified in outbreak investigations may not be representative of foods responsible for sporadic disease. Although a study found that outbreak and sporadic infections caused by four priority pathogens (*Salmonella*, *Campylobacter*, STEC O157 and *Listeria monocytogenes*) were similar in the USA, a number of published studies have noted that the food sources for some pathogens can vary substantially [16, 26, 44]. For STEC, potential differences are relevant for sources that are frequently involved in outbreaks (raw produce, unpasteurised dairy products), but are less likely to cause sporadic cases, either because contamination events are rare (even if with a large impact) or because they are not consumed frequently by the general population, but at high frequency among specific risk groups. To assess these differences, comparing outbreak data-driven estimates with source attribution estimates obtained with analysis of data from sporadic infections is paramount.

Our study did not adjust for older data (i.e. discounted or reduce the weight of older outbreaks) like other studies have done [44] because data were sparse and discounting data would lead to a further reduction of available information. To minimise potential bias introduced by large outbreaks, we also chose not to adjust for outbreak size. Though 4% of outbreak reports with known source included in this study involved greater than 100 cases, these outbreaks contributed 56% of associated cases. These very large outbreaks do not necessarily reflect the frequency of STEC contamination, but instead major contributing factors such as large-scale distribution and consumption as ready-to-eat foods [46–48]. Although our approach can also introduce bias and artificially reduce the relative importance of foods that frequently cause many (outbreak-related) cases, it provides confidence on the validity and utility of analysis of data from outbreaks to attribute all foodborne illnesses by a pathogen (i.e. sporadic and outbreak cases).

The source attribution method applied in this study attributes illness at the point of exposure/consumption and does not address the point in the farm to fork continuum where contamination occurred or was amplified. We have also focused only on foodborne outbreaks and thus did not investigate the role of other transmission routes (e.g. environmental, direct contact with animals) in STEC illnesses. Other source attribution methods attribute illnesses at the origin of the pathogen and/or investigate different transmission routes from the same origin [14]. Even though we acknowledge the advantages of such methods to estimate the relative importance of sources and exposure routes for foodborne infections, we concluded that attributing STEC illnesses at regional and global level was only feasible by applying point of exposure methods. A recent study attributed STEC infections to sources in the Netherlands, using a combined approached that allowed identification of the most important livestock sources of the pathogen and associated risk factors [5]. It showed that risk factors for STEC infection may vary according to the attributed source and thus provided an approach for generating hypotheses on the transmission pathways for STEC. While providing important supporting evidence that a growing number of unusual vehicles are associated with human STEC infections, it is not possible with the available data to apply this approach at a regional or global level.

Although foodborne outbreaks receive media and political attention, the main part of the burden of foodborne diseases consists of sporadic cases. Thus far, few countries have implemented surveillance of sporadic cases of foodborne disease, particularly in the developing world, where the majority of reported human cases are associated with foodborne outbreaks. In general, outbreak data have the advantage of being widely available worldwide, including countries or regions where sporadic cases of disease are not likely to be reported. However, the data obtained were limited and biased towards high income countries. The available data represented only three of the six WHO regions and even region representativeness may be questioned. Many countries did not report outbreak-associated cases, possibly due to either a lack of data reporting or a lack of outbreak surveillance. Extrapolation of our results to the global level should therefore be done with caution.

In general, the results of the outbreak analysis presented here and the estimates of the expert elicitation conducted by FERG were largely in coherence [10]. Differences between outbreak and expert elicitation estimates could be explained by the fact that the expert elicitation was not limited to outbreaks (i.e. experts were asked to estimate attribution proportions for all cases, sporadic and outbreak-associated) and because limited evidence on the relative contribution of different sources for STEC illness were available to inform the expert's estimations. We estimated regional STEC source attribution proportions for the regions for which data were available. Data were lacking from three WHO regions: African, South-East Asian and Eastern Mediterranean. Within some of the regions with available data, the number of countries that investigated and reported STEC outbreaks was limited (particulary AMR and WPR), which influences the representativeness of regional results. Improvement of surveillance and data collection is paramount to improve the representativeness of results. We expect that as outbreak investigation and surveillance capacity across the world increases, this approach will prove more useful for source attribution of STEC at global, regional and local levels.

## Conclusion

The most important sources of STEC infections are beef, produce and dairy, with regional variability in source attribution proportions. Despite data gaps, particularly in three WHO regions, these results suggest that targeting these sources when developing food safety interventions will have the greatest impact in reducing the burden of STEC foodborne disease. Further risk assessments are needed to support regulatory requirements, ideally building on novel detailed and country specific data.

## Supplementary material

For supplementary material accompanying this paper visit https://doi.org/10.1017/S095026881900116X. click here to view supplementary material
