# Supplementary material for: Global and regional source attribution of Shiga toxin-producing Escherichia coli infections using analysis of outbreak surveillance data
Source: Epidemiol Infect. 2019 Jul 8;147:e236. doi: 10.1017/S095026881900116X (PMC6625198; doi:10.1017/S095026881900116X)
Supplement: Supplementary file 1 [file S095026881900116Xsup001.zip › S095026881900116Xsup001/Supplementary material 1.docx]

#

# Supplementary material 1. Call for data launched by the World Health Organization.

#
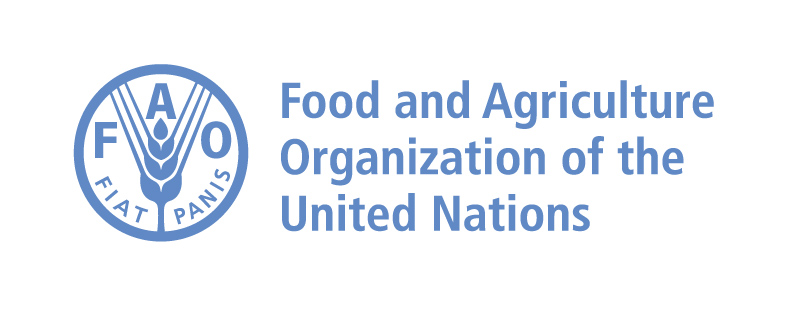

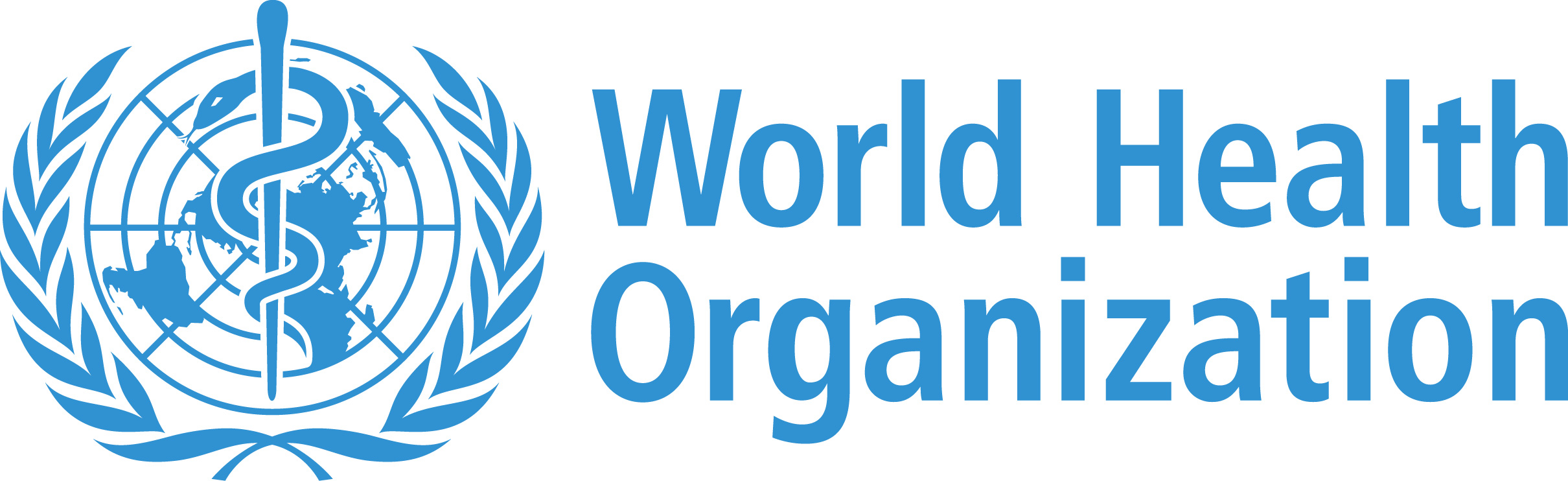


**Joint FAO/WHO Expert Meetings on Microbiological Risk Assessment (JEMRA)**

# CALL FOR DATA FOR SOURCE ATTRIBUTION AND MONITORING PROGRAMS FOR SHIGA TOXIN-PRODUCING *E. COLI* (STEC)

## Background

Verotoxigenic *Escherichia coli* (VTEC) / Shiga toxigenic *E.coli* (STEC) are an important cause of foodborne disease. Infections have been associated with a wide range of symptoms from mild intestinal discomfort to haemolytic uremic syndrome (HUS), end-stage renal disease (ESRD) and death. In its report on the global burden of foodborne disease, WHO estimated that foodborne STEC caused more than 1 million illnesses, 128 deaths, and nearly 13,000 DALYs2 in 2010^[[1]](#footnote-1)^.

The Codex Committee on Food Hygiene (CCFH) has discussed the issue of STEC in foods since its 45th Session and at the 47th Session, November 2015, it was agreed that it was an important issue to address (REP 16/FH, 2015). To facilitate this work, the CCFH requested FAO and WHO to develop a report compiling and synthesizing available relevant information, using existing reviews where possible, on the following aspects of STEC:

1. The global burden of disease and source attribution;
2. Hazard identification and characterization of STEC; and
3. Current monitoring and assurance programs including the status of the currently available methodology (commercially available and validated for regulatory purposes) for monitoring of STEC in food as a basis for management and control.

A meeting of a joint FAO/WHO Core Expert Group on VTEC/STEC was held in Geneva, Switzerland, from 19-22 July, 2016. A report^[[2]](#footnote-2)^ on the scope of the work and the approaches and the methodologies that may be used based on the experts' knowledge and experience was developed and presented to the Codex committee at its 48^th^ session in Los Angeles from 7 to 11 November, 2016.

On the monitoring and assurance programs, limited information was obtained from some regions in response to the call for data. For source attribution, more data is needed to estimate the proportion of STEC illnesses caused by specific food commodities. The CCFH encouraged countries to provide more data, and agreed on the recommendations of the core expert meeting to issue a targeted call for data especially on monitoring and quality assurance programs. FAO and WHO are requiring data on the following aspects:

## Objectives

The data will serve as inputs to the development of a report on the currently available monitoring and assurance programs in member countries and a report on source attribution for STEC illnesses at global and regional level. This call is supplemental to the earlier calls issued earlier in 2016^[[3]](#footnote-3)^ and is being issued in response to request of the CCFH.

## Request for relevant information

FAO and WHO want to ensure that all available and relevant information/data are collected, and are requesting governments, the food industry, academia, consumer groups, laboratories, health care providers and any other interested organizations and individuals to submit any available data from public health surveillance and assurance programs for STEC. These data may be published or unpublished. Reference should be made to related published studies, where applicable.

FAO and WHO also recognize that countries may be at different levels in the development of their monitoring programs and testing methods for STEC and welcome information on the status including challenges encountered.

## Confidential and/or unpublished data

FAO and WHO recognize that some of the information and relevant data which are now required may be unpublished or of a confidential nature. With regard to unpublished information and data, this remains the property of the author for subsequent publication by the owner as original material. Unpublished confidential studies that are submitted will be safeguarded in so far as it is possible to do so without compromising the work of FAO and WHO. Specific issues relating to confidentiality should be discussed directly between the information and data owners and FAO/WHO. For these and other issues please contact FAO and WHO at the contacts provided.

## Correspondence

For more information, please contact Dr Blaise Ouattara (FAO) or Dr Rei Nakagawa (WHO).

| Office of Food Safety  Attention: **Dr Blaise Ouattara**  Food and Agriculture Organization of the United Nations,  Viale delle Terme di Caracalla  00153 Rome, Italy  *Telephone*: + 39 06 5705 5636 *Email*: [jemra@fao.org](mailto:jemra@fao.org) | And | Department of Food Safety and Zoonoses  Attention: **Dr Rei Nakagawa**  World Health Organization 20, avenue Appia  1211 Geneva 27  Switzerland  *Telephone*: +41 22 791 3640 *Email*: [jemra@who.int](mailto:jemra@who.int) |
| --- | --- | --- |

The principle of source attribution by analysis of outbreak data is to estimate the relative contribution of different food sources to illness by a pathogen on the basis of the number of outbreaks and outbreak-cases that were caused by each food. The approach that will be used to extrapolate the results of outbreak investigations to estimate the sources of illness in the population will be able to include data on outbreaks caused by simple/single-ingredient foods (such as beef), and by complex/multi-ingredient foods (e.g. hamburger sandwich).

To be able to apply the approach at a global and regional level, it is important to gather data from all regions and (if possible) all countries. Harmonization of data fields will facilitate the data compilation process.

Data requirements are as follow:

- Data overview: number of STEC-outbreaks reported in the country, number of investigated outbreaks, number of outbreaks for which the causative source was identified.
- For each outbreak reported, information on:
  - Country, region*
  - Date*
  - Pathogen subtype
  - Number of suspected and confirmed cases*
  - Number of hospitalizations
  - Number of deaths
  - Number of complications (e.g. HUS)
  - Setting (e.g. home, restaurant, institution (type))*
  - Implicated food*
  - Level of evidence (laboratory, epidemiological)*
  - Trace back of contamination point

*Essential information

1. World Health Organization, 2015. WHO estimates of the global burden of foodborne diseases Available at <http://www.who.int/foodsafety/areas_work/foodborne-diseases/ferg/en/> [↑](#footnote-ref-1)
2. Joint FAO/WHO core expert group meeting on VTEC/STEC, Geneva Switzerland, 19-22 July, 2016 – Meeting report. Available at <http://www.fao.org/3/a-bq529e.pdf> and <http://www.who.int/foodsafety/areas_work/microbiological-risks/JEMRA-report.pdf?ua=1> [↑](#footnote-ref-2)
3. Call for data on Verotoxigenic (VTEC)/Shigatoxigenic(STEC). Available at <http://www.fao.org/3/a-bl019e.pdf> [↑](#footnote-ref-3)
