# Supplementary material for: Global and regional source attribution of Shiga toxin-producing Escherichia coli infections using analysis of outbreak surveillance data
Source: Epidemiol Infect. 2019 Jul 8;147:e236. doi: 10.1017/S095026881900116X (PMC6625198; doi:10.1017/S095026881900116X)
Supplement: Supplementary file 1 [file S095026881900116Xsup001.zip › S095026881900116Xsup001/Supplementary material 2.docx]

Supplementary material 2. Total number of STEC outbreaks reported per country and World Health Organization (WHO) Region*

| **Country** | **Region** | **Total** |
| --- | --- | --- |
| Argentina | AMR | 18 |
| Australia | WPR | 23 |
| Austria | EUR | 8 |
| Belgium | EUR | 10 |
| Canada | AMR | 54 |
| Croatia | EUR | 2 |
| Denmark | EUR | 9 |
| Finland | EUR | 2 |
| France | EUR | 59 |
| Germany | EUR | 9 |
| Hong Kong | WPR | 3 |
| Hungary | EUR | 1 |
| Ireland | EUR | 10 |
| Japan | WPR | 6 |
| Luxembourg | EUR | 1 |
| Malta | EUR | 1 |
| Netherlands | EUR | 4 |
| New Zeland | WPR | 3 |
| Norway | EUR | 3 |
| Poland | EUR | 4 |
| Portugal | EUR | 2 |
| Romania | EUR | 1 |
| Slovakia | EUR | 1 |
| Spain | EUR | 6 |
| Sweden | EUR | 13 |
| United Kingdom | EUR | 30 |
| US | AMR | 674 |
| **Total** |  | **957** |

*AMR: Region of the Americas; EUR: European region; WPR: Western Pacific region.
