# Supplementary material for: Global and regional source attribution of Shiga toxin-producing Escherichia coli infections using analysis of outbreak surveillance data
Source: Epidemiol Infect. 2019 Jul 8;147:e236. doi: 10.1017/S095026881900116X (PMC6625198; doi:10.1017/S095026881900116X)
Supplement: Supplementary file 1 [file S095026881900116Xsup001.zip › S095026881900116Xsup001/Supplementary material 3_.docx]

**Supplementary material 3.** Proportion of STEC cases attributed to foods based on the analysis restricted to outbreaks that involved HUS cases and to outbreaks that involved deaths (%, mean and 95% uncertainty interval [UI]). Analysis restricted to the American region.

|  | **Outbreaks with HUS cases** | | | **Outbreaks without HUS cases** | | | **Outbreaks with deaths** | | | **Outbreaks without deaths** | | |
| --- | --- | --- | --- | --- | --- | --- | --- | --- | --- | --- | --- | --- |
|  | **Mean** | **95% UI** | | **Mean** | **95% UI** | | **Mean** | **95% UI** | | **Mean** | **95% UI** | |
| **Eggs** | 0 |  |  | 0 |  |  | 0 |  |  | 0 | 0 | 0.1 |
| **Dairy** | 7.6 | 7.5 | 8.3 | 4.6 | 4.2 | 5 | 7.1 | 7.1 | 7.1 | 5.4 | 5.1 | 5.8 |
| **Meat** | 2.2 | 2.2 | 2.6 | 0.6 | 0.6 | 0.8 | 2.4 | 2.4 | 2.4 | 1 | 1 | 1.3 |
| **Poultry** | 0 |  |  | 0 |  |  | 0 |  |  | 0 |  |  |
| **Chicken** | 0 |  |  | 0.2 | 0.2 | 0.4 | 0 |  |  | 0.2 | 0.1 | 0.3 |
| **Ducks** | 0 |  |  | 0 |  |  | 0 |  |  | 0 |  |  |
| **Turkey** | 0 |  |  | 0 |  |  | 0 |  |  | 0 |  |  |
| **Beef** | 17.6 | 16.7 | 18.7 | 18.6 | 18.1 | 18.9 | 20.1 | 19 | 21.4 | 18.2 | 17.8 | 18.5 |
| **Pork** | 0.4 |  |  | 1.6 | 1.4 | 1.9 | 0 |  |  | 1.3 | 1.1 | 1.6 |
| **Lamb** | 0.4 |  |  | 0.4 | 0.4 | 0.6 | 0 |  |  | 0.5 | 0.4 | 0.6 |
| **Mutton** | 0 |  |  | 0 |  |  | 0 |  |  | 0 |  |  |
| **Game** | 0.4 | 0.4 | 0.4 | 0.6 | 0.6 | 0.8 | 0 | 0 | 0 | 0.6 | 0.6 | 0.7 |
| **Produce** | 16.3 | 15.4 | 17.1 | 16 | 15.3 | 16.6 | 20.4 | 19 | 21.4 | 15.8 | 15.3 | 16.3 |
| **Grains and beans** | 0.9 | 0.9 | 1.3 | 1.6 | 1.2 | 2.1 | 0 | 0 | 0 | 1.5 | 1.1 | 2 |
| **Seafood** | 0.4 |  |  | 0.4 |  |  | 0 |  |  | 0.4 |  |  |
| **Nuts** | 0.4 |  |  | 0.4 |  |  | 2.4 |  |  | 0.3 |  |  |
| **Oils and sugar** | 0 |  |  | 0 |  |  | 0 |  |  | 0 |  |  |
| **Unknown** | 53.1 |  |  | 55 |  |  | 47.6 |  |  | 54.8 |  |  |
